# Supplementary material for: Molecular Motors Orchestrate Pause-and-Run Dynamics to Facilitate Intracellular Transport
Source: Biomolecules. 2026 Feb 2;16(2):221. doi: 10.3390/biom16020221 (PMC12937800; doi:10.3390/biom16020221)
Supplement: Supplementary file 1 [file biomolecules-16-00221-s001.zip › Shen_SuppInfo.pdf]

Molecular motors orchestrate pause-and-run dynamics to facilitate intracellular transport

Yusheng Shen[1] and Kassandra M. Ori-McKenney[1,*]

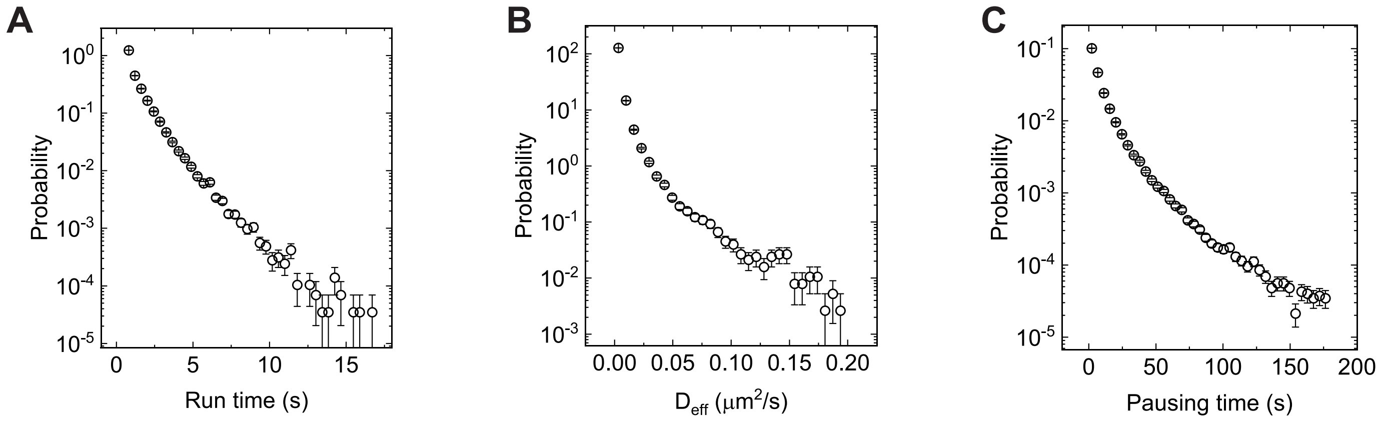

Figure S1. Pausing state of Rab5-positive endosome transport is vastly heterogeneous. (A) Measured PDF of the dwell time for run segments extracted from mobile trajectories. Total segments analyzed are n = 70451. (B and C) Measured PDF of the effective diffusion coefficient D_{eff} (B) and dwell time (C) in the pausing state for Rab5-positive endosomes. Total pausing segments analyzed from the mobile fraction are n = 84165.

Movie S1. Single-molecule dynamics of KIF5B(a.a. 1-560)-mStayGold in a BEAS-2B cell. A 2 min movie of the dynamics of KIF5B(a.a. 1-560)-mStayGold in BEAS-2B cells (5 fps). Single KIF5B(a.a. 1-560)-mStayGold motors exhibited unidirectional, processive motility along linear tracks without pausing, showing some preferential movement along specific microtubules. Scale bar, 2 μm.

Movie S2. Rab6A-positive secretory vesicle dynamics in a BEAS-2B cell. A 3 min movie showing the anterograde transport of Rab6A-positive secretory vesicle in BEAS-2B cells (10 fps). Rab6A vesicles switched between short, directed runs and longer, seemingly stalled, diffusive pauses. Scale bar, 5 μm.

Movie S3. Rab5-positive early endosome dynamics in a BEAS-2B cell. A 3 min movie showing the retrograde transport of Rab5-positive early endosome in BEAS-2B cells (10 fps). Rab5 endosomes switched between short, directed runs and longer, seemingly stalled, diffusive pauses. Scale bar, 5 μm.
